# Outsourcing Memory to External Tools: A Review of ‘Intention Offloading’

**DOI:** 10.3758/s13423-022-02139-4

**Published:** 2022-07-05

**Authors:** Sam J. Gilbert, Annika Boldt, Chhavi Sachdeva, Chiara Scarampi, Pei-Chun Tsai

**Affiliations:** Institute of Cognitive Neuroscience, 17 Queen Square, London, WC1N 3AZ UK

**Keywords:** Metamemory, Memory, Metacognition, Prospective memory, Cognitive offloading

## Abstract

How do we remember delayed intentions? Three decades of research into prospective memory have provided insight into the cognitive and neural mechanisms involved in this form of memory. However, we depend on more than just our brains to remember intentions. We also use external props and tools such as calendars and diaries, strategically placed objects, and technologies such as smartphone alerts. This is known as ‘intention offloading’. Despite the progress in our understanding of brain-based prospective memory, we know much less about the role of intention offloading in individuals’ ability to fulfil delayed intentions. Here, we review recent research into intention offloading, with a particular focus on how individuals decide between storing intentions in internal memory versus external reminders. We also review studies investigating how intention offloading changes across the lifespan and how it relates to underlying brain mechanisms. We conclude that intention offloading is highly effective, experimentally tractable, and guided by metacognitive processes. Individuals have systematic biases in their offloading strategies that are stable over time. Evidence also suggests that individual differences and developmental changes in offloading strategies are driven at least in part by metacognitive processes. Therefore, metacognitive interventions could play an important role in promoting individuals’ adaptive use of cognitive tools.

## Introduction

An essential prerequisite for living an independent and purposeful life is the ability to form, and subsequently fulfil, intentions. Intentions are mental representations of future goals and actions, which form part of the causal mechanism by which those goals and actions are eventually fulfilled or executed (Bratman, 1987; Grünbaum & Kyllingsbæk, 2020; Mele, 1992; Pacherie & Haggard, 2010; Searle, 1983). However, as everyone knows, intentions often go unfulfilled. This can be for many reasons, such as a lack of opportunity (Dholakia & Pbagozzi, 2003), motivation (Cook et al., 2015), or reprioritisation (Marsh et al., 1998). But a prominent cause of intentions going unfulfilled is a failure of memory. Everyone has had the experience of intending to do something, but finding that it has ‘slipped your mind’. Indeed, diary studies suggest that 50–70% of everyday memory failures reflect a failure to remember delayed intentions, rather than other forms of memory such as remembering facts or names (Crovitz & Daniel, 1984; Terry, 1988). This raises an obvious question: what are the mechanisms by which we remember delayed intentions, and how might these mechanisms be facilitated so that we can fulfil our intentions more effectively?

These questions have been addressed by studying ‘prospective memory’. More than three decades of research have delivered insights into the cognitive mechanisms by which we remember intentions, their underlying neural correlates, and the way that they change across the lifespan, in neurodevelopmental conditions, and in cases of brain damage or disease (for reviews, see Brandimonte et al., 1996; Cohen & Hicks, 2017; Kliegel et al., 2008b; McDaniel & Einstein, 2007; Rummel & McDaniel, 2019; Scullin et al., 2015). Much of this research has been based on experimental or laboratory tasks in which participants are first asked to remember one or more intentions, then their ability to fulfil these intentions is measured. For example, participants might be asked to perform an ongoing task such as sorting stimuli into words versus non-words, while also remembering a prior intention to press a third button when a predefined target word appears (Einstein & McDaniel, 1990). These studies help to explain the brain-based mechanisms by which individuals remember intentions. But we use more than just our brains to remember intentions. We also rely on external props and tools such as diaries, calendars, sticky notes, strategically placed objects, other individuals, and – increasingly – digital technologies such as alerts on smartphones or wearable devices. These are all examples of ‘intention offloading’ – the process of creating a cue in the external environment to trigger a delayed intention. As a result of intention offloading, intentions are stored in our extended physical and social environments, not just in our brains. However, while the brain-based mechanisms for remembering intentions have been extensively studied in recent decades, these extended mechanisms have received far less attention.

The purpose of this review is to summarise a line of research over the last 5–10 years seeking to investigate intention offloading. This is an example of the broader phenomenon of cognitive offloading, which has been defined as the use of physical action to alter the information processing requirements of a task so as to reduce cognitive demand (Risko & Gilbert, 2016). Everyday examples of cognitive offloading include diverse phenomena such as external normalization (tilting one’s head in order to perceive a rotated image, reducing the need for mental rotation; Risko et al., 2014); using a GPS device instead of internal memory to guide navigation (Brügger et al., 2019); storing to-be-remembered information in written form (Kelly & Risko, 2019; Lu et al., 2020; Risko & Dunn, 2015), on computers (Morrison & Richmond, 2020; Storm & Stone, 2014) or via photography (Barasch et al., 2017; Henkel, 2014; Soares & Storm, 2018); or searching for information on the internet via a search engine, rather than in internal memory (Ferguson et al., 2015; Fisher et al., 2015; Hamilton & Yao, 2018; Marsh & Rajaram, 2019). Here, we focus on the specific phenomenon of intention offloading; for a more general review, see Risko and Gilbert (2016).

We argue that this line of research is useful on both a theoretical and a practical level. On a theoretical level, the phenomenon of intention offloading is an example of ‘extended’ or ‘scaffolded’ cognition (Clark & Chalmers, 1998; Heersmink & Sutton, 2020; Hutchins, 1995; Kirsh, 1996). It provides a testbed for understanding the interrelations between numerous cognitive processes that are typically studied in isolation, such as memory, planning, metacognitive monitoring and control, and decision making. It also allows us to investigate how these cognitive processes vary between individuals, across the lifespan, and in neurodevelopmental conditions. On a practical level: intention offloading *works.* As discussed below, external reminders can have a large impact on whether or not individuals fulfil their delayed intentions. By understanding the mechanisms of intention offloading, we can aim to improve individuals’ adaptive use of cognitive tools.

Previous experimental studies have manipulated whether reminders are provided or not, as a means of measuring how much, if at all, they improve performance (Guajardo & Best, 2000; Guynn et al., 1998; Henry et al., 2012; Kliegel & Jäger, 2007; Lourenço & Maylor, 2015; Mahy et al., 2018; Meacham & Colombo, 1980; Ryder et al., 2022; Vortac et al., 1995). Using this approach, researchers can investigate the impact of experimenter-provided reminders on behaviour. This is important for understanding the relative efficiency of different types of reminders. For example, reminders of the prospective memory cue (when do I need to act?) may be more effective than reminders of the associated action (what do I need to do?; Ryder et al., 2022). However, very few experiments have given participants a free choice about whether or not to set reminders and then measured the strategy that participants choose (for two exceptions, see Einstein & McDaniel, 1990; Maylor, 1990). Therefore, while some studies have investigated the *consequences* of reminders, very few studies have systematically explored the *causes* of reminder-setting. The latter question will be the main subject of this review.

Our review is organised as follows. First, we address issues of measurement: How can intention offloading be measured, and what does this reveal about individuals’ preference or bias towards using external reminders versus internal memory processes? Second, what are the processes that trigger intention offloading? We consider the influence of metacognition, effort-avoidance and strategy perseveration. Third, how does intention offloading change across the lifespan, in child development and older age? Fourth, how does intention offloading relate to underlying brain mechanisms? Finally, we discuss some practical implications and suggest some promising directions for future research.

## Measuring Intention Offloading

A key step in advancing our understanding of prospective memory came from the development of paradigms that allowed it to be studied in the laboratory or clinic. Prominent approaches include the ‘Einstein-McDaniel’ procedure (Einstein & McDaniel, 1990), the ‘virtual week’ paradigm (Rendell & Craik, 2000), neuropsychological instruments such as the multiple errands test and six element test (Shallice & Burgess, 1991), the hotel test (Manly et al., 2002), the breakfast task (Craik & Bialystok, 2006) and clinical tools such as CAMPROMPT (Wilson et al., 2005). Without exception, these paradigms require participants to remember delayed intentions without assistance from external tools or reminders. Indeed, laboratory studies of prospective memory have tended to consider intention offloading as a source of unwanted noise that obscures ‘real’ prospective memory processes (Uttl & Kibreab, 2011). However, an alternative viewpoint is that intention offloading plays a key role in our ability to fulfil intentions in everyday life. So if we are to understand the processes by which intentions are actually fulfilled, then intention offloading needs to be considered alongside purely brain-based mechanisms rather than being considered as a nuisance. But how can intention offloading be measured experimentally?

**Fig. 1:**
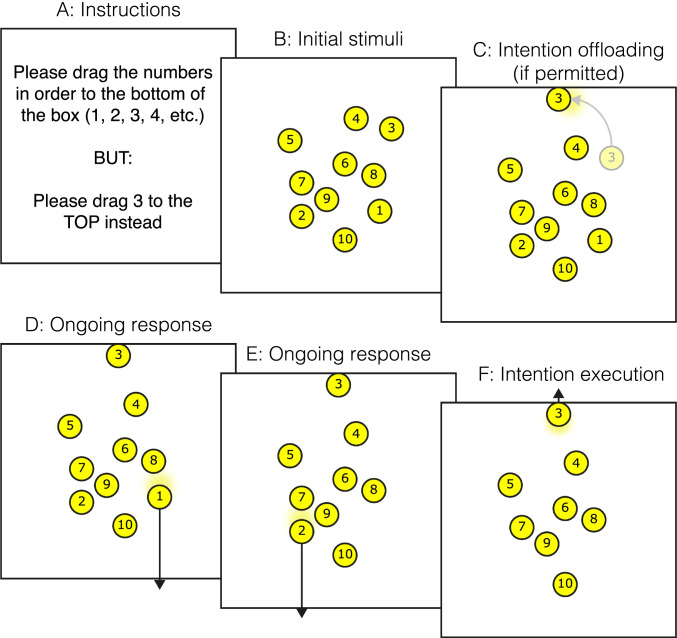
Schematic illustration of the intention offloading task

Gilbert (2015a) developed an experimental paradigm for this purpose that could be administered as an online web-based experiment (see Fig. 1). Participants are instructed to drag ten numbered circles in numerical sequence to the bottom of a box shown on the screen. Delayed intentions are embedded within this task, by presenting instructions at the beginning of each trial, for example ‘please drag number 3 to the top instead’. Therefore, participants are asked to produce a nonstandard response when they encounter a specific future cue. If they forget the intention, they can produce a standard ongoing response instead (dragging the circle to the bottom of the screen) and there is no physical characteristic of the target circles that directly cues their status as special items that require a nonstandard response. These characteristics match standard laboratory prospective memory paradigms such as the Einstein-McDaniel procedure. However, whereas previous studies of prospective memory typically impose retention intervals of minutes or longer between participants encoding intentions and acting on them, here the retention interval is on the order of seconds.

There are two ways in which participants performing this paradigm can remember intentions. They can remember the instructions with brain-based memory processes. Alternatively, they can set reminders by immediately dragging target circles next to their instructed location at the beginning of the trial, for example dragging the ‘3’ circle next to the top border of the box as soon as the trial begins, so that its location will remind the participant of the instruction when they get to this number in the sequence. This can be considered as analogous to placing an object next to your front door so that you remember to bring it with you when leaving the house tomorrow. In experiments using this paradigm, participants are typically instructed about this strategy and told that they have a free choice about whether to set reminders in this manner or simply remember intentions using their own internal memory processes. The rate of reminder setting (i.e., the proportion of target circles for which participants set up reminders in this way) can then be measured.

Gilbert (2015a) reported three main findings. First, participants were much more likely to set reminders when they had three items to remember rather than just one. Second, participants were more likely to set reminders when they encountered interruptions during the task (a pop-up box asking an arithmetic verification question). Therefore, intention offloading was influenced by both the memory load and the nature of the ongoing task in which this memory load was embedded. Third, the conditions associated with greater intention offloading (higher memory load or greater interruption) were also associated with reduced accuracy when participants were forced to use internal memory alone. This suggests that individuals set external reminders in order to mitigate failures of internal memory that might otherwise occur.

An additional purpose of Gilbert (2015a) was to investigate the relationship between participants’ performance of the experimental task and their fulfilment of a naturalistic intention embedded within their everyday life over a longer time period. Participants were provided with a unique web-link and told that they could earn additional small bonus payments by visiting this link after 2 days, 5 days and 7 days. For those participants who initially reported that they intended to collect all three bonuses, it could then be calculated whether there was a correlation between (a) the number of bonuses they actually remembered to collect over a 1-week period, and (b) accuracy on the experimental tasks in the initial testing session. There was a numerically small but statistically highly significant correlation between performance of the intention offloading task (with a retention interval of a few seconds) and the naturalistic task (with a retention interval of up to a week). The intention offloading task had greater predictive validity for the naturalistic task than any of the other measures that were examined, including more traditional event- and time-based prospective memory tasks.

This finding speaks to the question of whether it is appropriate to consider the intention offloading task as a task of prospective memory. An alternative viewpoint would be that the intention offloading task is a measure of short-term or working memory, but does not qualify as a task of prospective memory because the retention interval is so short. According to this account, the term ‘prospective memory’ should be reserved for tasks involving a longer retention interval so that participants cannot continuously rehearse their intended action, but instead need to bring it back to mind after a delay (Graf & Uttl, 2001). In our view, this question of terminology is somewhat arbitrary. It is undoubtedly the case that the intention offloading task requires participants to form delayed intentions and then fulfil them after a delay. It is also clearly true that the task has a shorter retention interval than typical prospective memory paradigms. Nevertheless, the task has some external validity in the sense that performance predicts participants’ fulfilment of a real-world intention over a 1-week period, with a greater predictive validity than more traditional prospective memory tasks. This remains the case regardless of whether it is labelled as a prospective memory task or some other sort of task, and none of the conclusions drawn below are affected by this terminological question. To avoid this issue, we generally prefer to use the more neutral term ‘memory for delayed intentions’ rather than ‘prospective memory’, which some authors choose to use in a more restricted sense.

## Optimality of Intention Offloading

Setting a reminder involves both a cost (the time and effort setting it up) and a benefit (the increased chance of remembering). These costs would mount to an unacceptable level if we set reminders for absolutely every activity we intend to perform, including routine daily activities like remembering to go to work, to eat, to sleep, and so on. Therefore, individuals need to continually make decisions about whether the benefit of setting a reminder outweighs the cost. While the paradigm used by Gilbert (2015a) allows measurement of how often participants decide to set reminders, it cannot be used to determine how optimal these decisions are. To investigate this question, Gilbert et al. (2020) adapted the earlier paradigm so that it can be used to determine whether individuals weigh the costs and benefits of reminders optimally or show a systematic bias either towards or away from using them.

**Fig. 2:**
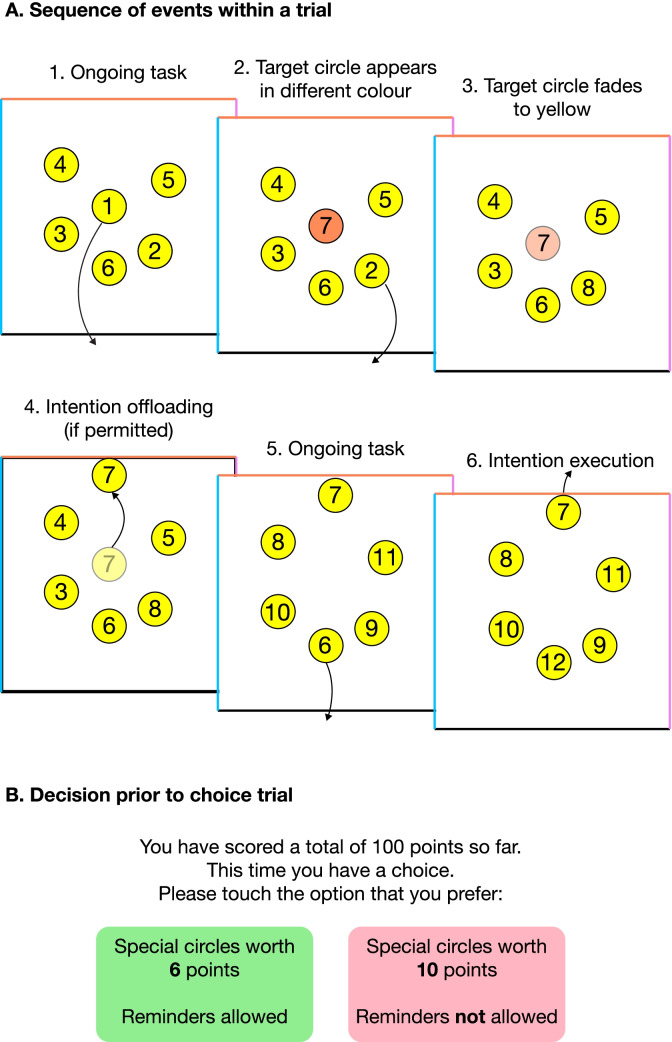
Schematic illustration of the ‘optimal reminders’ variation of the intention offloading task. Participants drag circles in numerical sequence to the bottom of the box, removing them from the screen. Each circle that is removed is replaced with another one that continues the sequence. Sometimes new circles appear in a colour that matches one of the other sides of the square. This represents an instruction to drag that circle to the corresponding side when it is eventually reached in the sequence. Multiple target circles are presented within a single trial, meaning that participants are unlikely to remember all of them if they rely only on internal memory. However, the task is straightforward if reminders are used

In this paradigm (Fig. 2), participants perform a demanding task, where mean accuracy using internal memory is typically around 50–60%. Alternatively, they can set external reminders, in which case accuracy is typically 90–100%. As they perform this task, they are repeatedly offered a choice between (a) using their own memory, in which case they earn maximum points for each remembered item, or (b) using reminders, in which case they earn a smaller number of points which varies from trial to trial. Suppose an individual can achieve 55% accuracy using their own memory and 100% using reminders. Offered a choice between 10 points per item using their own memory and 5 points using reminders, the optimal choice is to use internal memory (which would earn on average 5.5 points per item) rather than reminders (which would earn only 5 points). But if 6 points per item are offered using reminders, it becomes optimal to switch to this strategy instead.

On some trials, participants are forced to use either internal memory or reminders. Based on performance on these trials, the optimal strategy can be calculated for the choice trials. This can then be compared against the actual choice behaviour to evaluate whether participants use (a) more reminders than would be optimal, (b) fewer reminders than would be optimal, or (c) the optimal number of reminders. Note that calculation of this measure is individually tailored to each participant’s accuracy on the forced internal and external trials. So a particular reminder-setting strategy such as always setting reminders when offered 6 points or higher might reflect an under-use of reminders for a participant with relatively poor memory, but an over-use of reminders for a participant with relatively good memory. Studies using this paradigm have consistently found evidence for a systematic bias: Individuals tend to set reminders on a greater number of trials (and, equivalently, use internal memory on a smaller number of trials) than would be optimal (Ball et al., 2022; Engeler & Gilbert, 2020; Gilbert et al., 2020; Kirk et al., 2021; Sachdeva & Gilbert, 2020). Individual differences in this bias are stable over time (Gilbert et al., 2020, Experiment 1). Results also show that participants with poorer memory ability tend to set reminders on a greater number of trials than those with better memory ability (Gilbert et al., 2020).

## Metacognition as a Trigger for Intention Offloading

Each time we form an intention, we need to decide whether to remember it internally or set an external reminder. How do individuals make these decisions? One clue comes from the data described above (Gilbert, 2015a), showing that participants are increasingly likely to set reminders in conditions where their performance is poorer (i.e., with a higher memory load or interruptions during task performance). This could be explained by an influence of metacognition, our ability to monitor and control our own cognitive processes and abilities (Dunlosky & Metcalfe, 2008; Flavell, 1979; Fleming et al., 2012; Koriat, 2007; Metcalfe, 1996; Nelson & Narens, 1990). Given that individuals set more reminders in situations where their own memory processes tend to be inadequate, this suggests that they rely on a metacognitive evaluation of their own memory abilities to trigger intention offloading when it is necessary (see also Arango-Muñoz, 2013; Weis & Wiese, 2020).

Previous studies have investigated metacognition of prospective memory with a variety of approaches, including asking participants to make quantitative assessments of their performance (e.g., Cauvin et al., 2018; Meeks et al., 2007; Rummel et al., 2013; Schnitzspahn, Zeintl, et al., 2011) or via questionnaires (Crawford et al., 2006; Rummel et al., 2019). Results suggest that individuals do have some metacognitive awareness of their prospective memory abilities (Meeks et al., 2007), but they are often unduly pessimistic, that is, they predict that they will perform worse than they actually do (see Kuhlmann, 2019, for a review).

However, just because individuals tend to set reminders in conditions when internal memory is more likely to fail, this does not necessarily imply that they do so as a result of metacognitive processes. An alternative explanation could be that individuals learn by associative mechanisms the situations where intention offloading is most beneficial, or by earlier instruction to offload in particular situations, without directly engaging in metacognitive monitoring of their memory abilities. Direct evidence for a metacognitive influence on intention offloading would therefore require a demonstration that intention offloading is predicted by subjective metacognitive beliefs about memory ability, regardless of the need for offloading as measured by objective memory ability. Such evidence was found by Gilbert (Gilbert, 2015b; see also Dunn & Risko, 2016, for a conceptually related effect in a different domain of cognitive offloading). Participants performed an intention offloading task in two phases: first relying on internal memory only, second with the option of setting reminders if they wished. They also made metacognitive performance evaluations at each phase. Results showed that the likelihood of reminder-setting in phase 2 was predicted by objective unaided accuracy in phase 1 – how much they actually needed reminders, and, independently, by their metacognitive prediction in phase 1 – how much they *thought* they needed reminders. In one experiment (Gilbert, 2015b, Experiment 1a) participants’ use of reminders was predicted by metacognitive evaluations, even when those metacognitive evaluations were entirely unrelated to objective accuracy (r = -0.01), providing clear evidence for a metacognitive influence on intention offloading.

In another study (Boldt & Gilbert, 2019), one group of participants was explicitly instructed on how to set reminders if they wished to do so, similar to Gilbert (2015b). A second group was not provided with any instructions, so they could only set reminders if they spontaneously invented the strategy themselves. Results showed that participants in the spontaneous group did indeed spontaneously generate an offloading strategy; however, they did so to a lesser degree than the explicitly instructed group. Nevertheless, the same association with confidence was found in both groups: Participants with lower confidence were more likely to set reminders, regardless of objective memory ability. Therefore, even in a situation where the strategy needs to be spontaneously generated rather than explicitly instructed, intention offloading is still guided by low confidence (see also Hu et al., 2019).

Further evidence for a role of metacognition on intention offloading comes from a study where participants underwent metacognitive interventions, i.e., interventions designed to influence their metacognitive beliefs (Gilbert et al., 2020, Experiment 3). We manipulated the difficulty of practice trials and, independently, the feedback received. This feedback did not deceive participants but described the same level of performance in positive or negative terms, such as “Well done – excellent work! You responded correctly to most of the special circles” versus “Room for improvement. You got some of the special circles wrong”. Results showed that the metacognitive interventions influenced confidence: Participants were significantly more confident after receiving positive feedback, and when they received easy practice trials. However, there was no effect on objective accuracy. The metacognitive interventions also influenced reminder bias: To the extent that participants became more confident, they relied less on external reminders. Further, mediation analysis showed that shifts in reminder bias were mediated by shifts in confidence. Therefore, metacognitive interventions can affect reminder setting without affecting unaided memory performance, providing strong evidence for a metacognitive influence on intention offloading.

## Intention Offloading and the Avoidance of Cognitive Effort

As discussed in *Optimality of intention offloading* above, the optimal reminders paradigm typically reveals a positive reminder bias, that is, participants tend to rely on external reminders more than would be optimal. Consistent with a metacognitive account, they also tend to be underconfident, predicting lower accuracy than they actually achieve (Engeler & Gilbert, 2020; Gilbert et al., 2020). In this context a positive reminder bias is rational: Insofar as an individual believes that their internal memory processes are inadequate, they should rely on external resources instead. However, the metacognitive account cannot explain the reminder bias in full. In the metacognitive intervention study described above (Gilbert et al., 2020, Experiment 3), one group of participants who received both easy practice trials and positive feedback were significantly over-confident in their memory ability. The reminder bias in this group was significantly reduced, but it was nevertheless still positive, meaning that participants were still more likely to use external reminders than would have been optimal. If the reminder bias was wholly attributable to metacognitive bias, then just as an under-confident participant would be expected to over-rely on reminders, an over-confident participant would be expected to show a negative rather than a positive reminder bias. This study showed that a positive reminder bias can be observed in the context of both over- and under-confidence, indicating that one or more additional factors must be involved. Sachdeva and Gilbert (2020) provided evidence that one such factor is a preference to avoid cognitive effort.

The concept of cognitive effort is elusive to describe at a mechanistic level (Shenhav et al., 2017). Nevertheless, it is widely argued that cognitive effort is typically aversive (Dreisbach & Fischer, 2015; Kurzban, 2016; Saunders et al., 2017) and individuals generally tend to avoid effortful tasks (Frederick, 2005; Kool et al., 2010). Some theoretical accounts have proposed that cognitive effort is a limited, depletable resource that individuals strive to conserve (Baumeister et al., 2007). Although this viewpoint can potentially explain why individuals would avoid effortful tasks, it has encountered serious conceptual and empirical challenges (Hagger et al., 2016; Lurquin & Miyake, 2017). Contemporary theories of cognitive effort have focused on an alternative approach, arguing that the feeling of cognitive effort arises from the engagement of relatively domain-general processes that can only be deployed for a limited number of simultaneous tasks (Kurzban et al., 2013). Such domain-general processes incur an opportunity cost. In other words, exercising cognitive effort on one activity precludes its use on another. An aversion to cognitive effort can then be understood as a drive to reduce this opportunity cost.

This model can potentially explain both why individuals might avoid effortful tasks, and why remembering an intention with internal memory may be more effortful than using external reminders. There are well-known limits to the quantity of information that can be actively maintained in short-term or working memory (Bays & Husain, 2008; Miller, 1956). Maintaining one active intention therefore incurs an opportunity cost, since this may preclude simultaneously maintaining another one. By contrast, external tools such as smartphone alerts have an effectively unlimited capacity. As a result, individuals may prefer external reminders over internal memory because they incur a lower opportunity cost.

Sachdeva and Gilbert (2020) argued that manipulating performance-based financial rewards allows a test of whether effort-avoidance influences intention offloading. If excessive use of reminders is explained by metacognitive error alone, then it should not matter whether or not performance is incentivised with financial reward. Regardless of the reward, participants would be selecting the strategy that is optimal, based on their metacognitive beliefs. But if effort-avoidance makes an additional contribution, then the reminder bias should be reduced by financial incentives. Seeing as individuals are more likely to exert cognitive effort when they have a financial incentive to do so (Aarts et al., 2010; Padmala & Pessoa, 2011), we would predict that performance-based rewards would at least partially overcome the preference to avoid cognitive effort, and therefore the reminder bias would be reduced. In other words, a reduction of the reminder bias as a result of financial reward would be diagnostic of an influence of effort-avoidance, rather than metacognitive error. This is exactly what was found by Sachdeva and Gilbert (2020): the bias towards external reminders was significantly reduced, but not eliminated, when participants received a financial reward for their performance of the task. Therefore, a second factor contributing to individuals’ intention offloading decisions, in addition to metacognitive belief, is a preference to avoid cognitive effort.

## Strategy Perseveration

A third factor contributing to intention offloading was demonstrated by Scarampi and Gilbert (2020, Experiment 2). Participants performed an intention offloading task in two phases. In the first phase they were either forced to use reminders or forced to use their own memory. In the second phase they had a free choice about whether to set reminders. Results showed that despite their free choice of strategy, participants tended to perseverate with whichever strategy they had used in phase 1. Therefore, previous instruction or experience with an intention offloading strategy is an additional factor that influences ongoing offloading behaviour. Scarampi and Gilbert (2020, Experiment 1) also found that previous use of an offloading strategy did not influence subsequent unaided memory, at least in the short term.

These findings are relevant to the debate about the potential long-term benefits or harms of cognitive technology. It has been argued at least since the time of Socrates that relying on external tools rather than brain-based processes might lead to a harmful decline in cognitive ability (Kallick, 1989). This fear has found expression in contemporary debates about whether technologies such as Google are ‘making us stupid’ (Carr, 2008) or leading to ‘digital dementia’ (Moledina & Khoja, 2018). We consider that these fears are overstated because they hypothesise long-term harms based only on short-term evidence, and they disregard evidence showing that offloading can have positive as well as negative cognitive impacts (Cecutti et al., 2021; Runge et al., 2019; Storm & Stone, 2014).

Furthermore, the fear that using cognitive tools will have harmful consequences presents only one side of the cost-benefit calculation. The other side is that *failing* to use cognitive tools may be harmful if such tools are available and useful. Evidence shows that external reminders are highly effective. For example, participants in Gilbert et al. (2020) had a forgetting rate of about 45% when using their own memory but only around 5% using external reminders; in other words, using reminders reduced the forgetting rate by almost an order of magnitude (see also Jones et al., 2021). In this context, an individual who decides against using reminders, for example an older adult who believes in the importance of ‘use it or lose it’ to maintain cognitive health, will be depriving themselves of an extremely effective and convenient tool, which can promote all the benefits that come with being able to fulfil one’s delayed intentions effectively. In addition, the strategy perseveration effect reported by Scarampi and Gilbert (2020) suggests that an earlier decision to forgo reminders can influence future strategy as well. This could have a particularly harmful impact in the context of cognitive decline, which makes the effective use of compensatory tools ever more important.

## Intention Offloading across the Lifespan: Child Development

When and how do children develop the ability to supplement their brain-based cognitive processes with external reminders? Redshaw et al. (2018) investigated this question in children aged approximately 7–13 years, using an intention offloading task similar to that of Gilbert (2015a) administered with a touchscreen tablet computer. As in Gilbert (2015b), the task was performed in two phases, first using unaided memory and second with the option to set reminders. There were two levels of difficulty (one item vs. three items to remember) and participants provided separate metacognitive predictions for each level of difficulty in each phase.

Older children (11+ years of age) offloaded strategically in the same way as adults (Gilbert, 2015a): they were much more likely to set reminders when they had more items to remember. But younger children (< 9 years) were equally likely to offload intentions, regardless of the memory load. Could this be explained by a lack of metacognitive knowledge in the younger children? If they failed to understand the increased likelihood of forgetting at the higher memory load, it would be unsurprising if they did not compensate for this with increased reminder-setting. However, results from the metacognitive judgements ruled this out: if anything, the younger children were more sensitive than the older children to the increased likelihood of forgetting at the higher memory load. Therefore, it seems that the younger children possessed the metacognitive *knowledge* that they were more likely to forget at the higher memory load, but they lacked the metacognitive *control* to translate this into strategic reminder-setting targeted at the more difficult trials. This distinction between metacognitive knowledge and control is consistent with prior evidence from the metamemory literature – for example, younger children can distinguish between easy and difficult items for a memory test, but only older children allocate more study time to the more difficult items (Dufresne & Kobasigawa, 1989; Lockl & Schneider, 2004; Masur et al., 1973).

Subsequent work has demonstrated strategic reminder setting in younger children. Bulley et al. (2020) investigated a short-term retrospective memory task, finding that once the offloading strategy was instructed, even children as young as 4 years selectively set reminders at a higher memory load. However, only older children set reminders when they had to devise the strategy themselves. These findings show that under the right circumstances, selective offloading strategies may be observed in very young children (see also Armitage et al., 2020; Armitage & Redshaw, 2021). However, the scenario studied by Redshaw et al. (2018) could have presented a particular challenge for young children. Some potential reasons for this might be the need to remember intentions prospectively, rather than a directly cued retrospective memory test, and the intermixing of the two difficulty levels on a trial-by-trial basis.

## Intention Offloading across the Lifespan: Ageing

At the other end of the lifespan, research has begun to investigate how intention offloading strategies may change in older age. This is particularly relevant because experimental studies have found evidence for a significant decline in older adults’ prospective memory performance compared with younger adults (Ihle et al., 2013; Kliegel et al., 2008a; Uttl, 2008). Experimental results are mixed, however, with various factors influencing age-related effects including the memory load (Ballhausen et al., 2017) and the importance of the prospective memory task (Hering et al., 2014). As well as being of theoretical interest for theories of cognitive ageing and metacognition, the question of age-related change in intention offloading has clear practical relevance for finding interventions to improve older adults’ fulfilment of intentions. This may help promote older adults’ behavioural independence and could play a particularly important role in health-related intentions such as remembering to take medication.

There are two reasons to consider that older adults might make particularly effective use of intention offloading strategies. First, some studies of metamemory and ageing suggest that older adults may have particularly low confidence in their memory abilities (Dobbs & Rule, 1987; Touron, 2015; Touron & Hertzog, 2004). As we have seen above, low confidence would be expected to translate into increased reminder-setting. Second, studies of the ‘age-related prospective memory paradox’ (Schnitzspahn, Ihle, et al., 2011) have shown that although older adults perform worse than younger adults in laboratory prospective memory tasks, they tend to perform as well, or better, than younger adults in a real-world setting. One potential explanation for this has been that older adults tend to make increased use of external reminders. But other than some tentative evidence presented by Gilbert (2015a), direct experimental support for this hypothesis has been lacking (Maylor, 2008; Phillips et al., 2008). We therefore conducted two studies investigating age differences in intention offloading strategies, expecting – incorrectly, as it turned out – that older adults would show an increased preference for intention offloading.

Scarampi and Gilbert (2021) administered a version of the intention offloading task used by Gilbert (2015a) to a group of younger (18–30 years) and older (65–84 years) participants. In separate trials, we manipulated memory load (one item; three items) and reminder-setting (not allowed; optional). Results showed that when intention offloading was not allowed, the older group performed significantly worse than the younger group, particularly at the higher memory load. When they were given the option to set reminders, older adults did so slightly (but not significantly) more often than younger adults. However, older adults still performed significantly worse on the task, despite the option to set reminders as a compensatory strategy. Therefore, it cannot be assumed that older adults will compensate fully for memory difficulties when given the opportunity (a similar pattern of results was found in adults with autism spectrum conditions in a study by Cherkaoui and Gilbert (2017)). Additionally, older adults were over-confident in their unaided ability to perform the task, whereas younger adults were well calibrated. Therefore, contrary to our expectations, older adults were more likely to be overconfident in their memory ability and did not fully compensate for impaired performance even when they were allowed to set reminders.

A follow-up study by Tsai et al. (2022) used the optimal reminders paradigm introduced by Gilbert et al. (2020) to investigate the *bias* towards or away from reminders, relative to the optimal strategy (see *Optimality of intention offloading* above). Older adults performed significantly worse when using unaided memory. They also were significantly more likely to set reminders when they were allowed. Two potential causes of this could be (a) an adaptive response to reduced memory ability, and/or (b) a change in older participants’ preference towards internal memory versus external reminders, relative to the optimal strategy. Results showed that even though older adults set numerically more reminders, they were in fact *less* biased towards external reminders than younger adults. In other words, relative to their level of performance, older adults were less likely to set reminders. But because their level of performance was lower, they still set numerically more reminders. Younger adults tended to use external reminders even when the optimal strategy would have been to use internal memory. Consistent with this, they were underconfident in their own memory abilities. By contrast, there was no significant bias towards or away from reminders in the older group, and nor were they underconfident in their memory ability.

In sum, the results of Scarampi and Gilbert (2021) and Tsai et al. (2022) show that (a) older adults cannot always be expected to compensate for memory difficulties with external memory aids, (b) insofar as older adults set more reminders than younger adults, this seems to be better explained by an increased *need* for reminders, rather than an increased *preference* for external memory support; (c) in terms of bias, older adults sometimes show a reduced preference for external reminders than younger adults, and (d) these findings may be explained by different levels of memory confidence between younger and older participants. Therefore, both in case of child development (see *Intention offloading across the lifespan: Child development*) and ageing (this section), developmental change in offloading strategies seem to be explained, at least in part, by changes in metacognitive processes. This conclusion would be stronger, however, if it were supported by longitudinal rather than cross-sectional evidence.

Metacognitive factors could explain sub-optimal offloading strategies in two distinct ways. On the one hand, an individual might offload sub-optimally because they use incorrect metacognitive beliefs to determine their strategy. This appears to be the case in older adults studied by Scarampi and Gilbert (2021), who were overconfident in their memory ability and did not fully compensate for impaired performance when reminder-setting was allowed. On the other hand, an individual might offload sub-optimally because they fail to use metacognitive beliefs at all. This appears to be the case in the youngest children studied by Redshaw et al. (2018), who knew that they were more likely to forget when they had more items to remember, but did not use this knowledge to inform their reminder-setting strategy.

## Neural Basis of Intention Offloading

How do the behavioural findings reviewed above relate to underlying brain activity? There are two separate issues that might be addressed here. First, what are the brain processes that trigger intention offloading? Second, what are the downstream effects on subsequent brain activity? Boldt and Gilbert (2022) developed a new paradigm to investigate the first question using functional magnetic resonance imaging (fMRI). Participants performed a series of trials where they were asked to remember a delayed intention. On some experimental blocks they were asked how confident they were that they would remember. This question corresponds to metacognitive monitoring: it assesses participants’ evaluation of their own mental states and abilities. On other experimental blocks they were asked how much they wanted a reminder, which could help them remember. Their answer to this question determined their likelihood of actually receiving a reminder. This question corresponds to metacognitive control: it assesses a form of behavioural regulation that is putatively triggered by a metacognitive process, because low confidence should translate into an increased desire for a reminder.

Using multivariate pattern analysis (Haynes & Rees, 2006; Weaverdyck et al., 2020), we first showed that we could ‘decode’ metacognitive monitoring and, separately, metacognitive control. That is, using a machine learning approach, we found that we could train a pattern classifier to detect the difference between a high-confidence versus a low-confidence brain (i.e., metacognitive monitoring) or, separately, the difference between a brain with high versus low desire for a reminder (i.e., metacognitive control). Next, we showed that we could cross-classify between the two. So a pattern classifier that had only ever been trained to classify high versus low desire for a reminder also accurately classified the difference between a brain with high versus low confidence, even though it was presented with novel data and had never been trained on this distinction. However, cross-classification was less reliable than decoding metacognitive knowledge or control separately. Therefore, results provided evidence at a neural level that intention offloading is driven by a metacognitive process. They also showed that metacognitive monitoring and control are associated with partially, but not fully, overlapping brain processes.

Once intention offloading has been triggered, how does it affect subsequent brain activity associated with remembering delayed intentions? This question was addressed by Landsiedel and Gilbert (2015), who asked participants to perform a version of the intention offloading task (Gilbert, 2015a) whilst undergoing fMRI scanning. On each trial, participants were either instructed to use their own memory or to set external reminders. They also performed a separate intention-free baseline condition. Compared with the baseline condition, the conditions requiring memory for intentions yielded signal change in two sets of brain regions. One set of regions, including lateral rostral prefrontal cortex, *increased* their activity while participants remembered delayed intentions. In previous work (Gilbert, 2011) this region was suggested to play a ‘content-free’ role in remembering delayed intentions. That is, it is involved in remembering that *something* needs to be done, but not the detailed content of the intention. A second set of regions, including medial rostral prefrontal cortex, *decreased* their activity while participants remembered delayed intentions. Despite the net reduction in overall activation, this region has previously been shown to contain information corresponding to the precise content of intentions (Gilbert, 2011; Momennejad & Haynes, 2012, 2013). Therefore, Gilbert (2011) argued that lateral rostral prefrontal cortex may maintain a content-free preparedness to act on an intention, accompanied by a mean increase in activation from baseline, while the precise content is represented in medial rostral prefrontal cortex, accompanied by a mean decrease in activation from baseline.

How might intention offloading affect activity in these two brain regions? Landsiedel and Gilbert (2015) argued that it is no longer necessary to store the precise content of an intention once this has been represented in an external store. Consistent with this, deactivation of medial rostral prefrontal cortex was attenuated once participants set reminders. By contrast, it may still be necessary to remember that *something* needs to be done, to maintain a preparedness to act on an intention even once the content has been stored externally. Consistent with this, there was no reduction in lateral rostral prefrontal cortex activity when participants set reminders. Therefore, intention offloading can have dissociable effects on brain regions thought to play distinct roles in remembering delayed intentions. Signal change associated with remembering that something needs to be done, without the precise content of the intention, may be relatively unaffected. By contrast, signal change associated with storing the content of an intention may be reduced.

## Practical Implications and Open Questions

We conclude by considering some of the practical implications of the work described above, and discussing open questions for future research. The first conclusion that we draw is that an individual’s level of performance on a cognitive task may relate at least as much to the cognitive strategies they choose as their unaided level of cognitive ability. Consistent with this, the availability of cognitive offloading strategies can attenuate individual differences related to unaided ability. Ball et al. (2022) found that individuals’ unaided ability to remember delayed intentions was associated with their working memory ability, measured in a separate task. But once intention offloading was permitted, there was no longer any relationship between task performance and working memory ability. This suggests that patterns of individual differences on real-world tasks, where various offloading strategies are typically available, may be different to those measured on laboratory tasks, where offloading is typically not allowed. However, the studies reviewed above were mostly conducted with rather artificial experimental tasks. We do not yet know much about how individuals’ cognitive offloading strategies, measured in the laboratory, relate to their use of cognitive offloading in everyday life. This may be particularly relevant to understanding the effect of ageing on memory, where findings have been inconsistent between laboratory and naturalistic settings (Cauvin et al., 2018; Devolder et al., 1990; Phillips et al., 2008; Schnitzspahn, Ihle, et al., 2011).

A second conclusion that may be drawn from the studies reviewed above is that forgetting is not necessarily the only source of variance, or even the predominant source of variance, influencing whether or not individuals fulfil their intentions. Another factor that may be at least as important in determining the fulfilment of intentions is whether or not they are considered sufficiently important to be offloaded into an external store.

This was investigated by Dupont et al. (in press), who showed that high-value intentions (associated with a larger financial reward) were much more likely to be offloaded than low-value intentions. One corollary of this is that individuals can potentially be left with nothing but low-value information in internal memory if the external store fails. These findings highlight the importance of understanding the factors that influence intention offloading, if we wish to understand intention fulfilment. This review has summarised evidence pointing towards multiple factors, which we list in Table 1. However, there are undoubtedly additional factors that have not yet been investigated fully, for example individual differences in personality or cognitive style (see Kirk et al., 2021), the influence of neurodevelopmental conditions (see Cherkaoui & Gilbert, 2017), and sociocultural effects.

**Table 1:** Factors influencing intention offloading

Factor	Finding	Evidence
Memory load	Higher memory load increases offloading	Gilbert (2015a), Scarampi and Gilbert (2020, 2021), Redshaw et al. (2018)
Task interruption	Task interruption increases offloading	Gilbert (2015a)
Memory confidence	Lower confidence is associated with increased offloading	Ball et al. (2022), Boldt and Gilbert (2019), Gilbert (2015b), Gilbert et al. (2020), Kirk et al. (2021), Engeler and Gilbert (2020)
Objective memory ability	Lower objective ability is associated with increased offloading	Gilbert (2015b), Gilbert et al. (2020). Though see also Boldt and Gilbert (2019).
Perceptual confidence	Lower confidence in perceptual judgements is associated with increased offloading in a separate memory task	Gilbert (2015b)
Metacognitive interventions: advice	Metacognitive advice reduces bias in reminder-setting	Gilbert et al. (2020, Experiment 2)
Metacognitive interventions: practice difficulty	Difficult practice trials increase subsequent offloading	Gilbert et al. (2020, Experiment 3)
Metacognitive interventions: feedback	Negative feedback increases offloading	Gilbert et al. (2020, Experiment 3), though see also Engeler and Gilbert (2020) and Grinschgl et al. (2020)
Financial reward (1)	Greater reward for intention fulfilment increases offloading	Dupont et al. (in press)
Gain versus loss framing	Bias towards offloading is reduced when it is framed in terms of loss rather than gain	Fröscher et al. (2022)
Financial reward (2)	Financial incentive leads to more optimal offloading (less effort avoidance)	Sachdeva and Gilbert (2020)
Task instructions	Explicit instructions increase uptake of offloading strategy	Boldt and Gilbert (2019)
Strategy perseveration	Prior use of offloading strategy predicts its continuation	Scarampi and Gilbert (2020)
Child development	Older age predicts greater selectivity of offloading strategy for higher memory loads	Redshaw et al. (2018)
Older age: absolute use of reminder	Older age predicts numerically increased use of reminders	Scarampi and Gilbert (2021); Tsai et al. (2022)
Older age: bias towards reminders	Older age predicts reduced bias towards reminders, relative to objective need	Tsai et al. (2022)

Our third and main conclusion is that intention offloading is a highly effective strategy for remembering delayed intentions, and metacognitive processes play a key role in triggering it. The studies reviewed above provide evidence that individual differences in offloading strategies, and developmental changes in those strategies, are driven at least in part by metacognitive processes. Therefore, metacognitive interventions could play an important role in promoting the adaptive use of cognitive tools. Insofar as individuals have a more accurate understanding of their true internal memory ability, they can target their use of external resources more effectively. We suggest, speculatively, that metacognitive interventions could have a greater impact on everyday functioning than ‘brain training’, which, despite being a billion dollar industry, seems to have a weak impact at best on general cognitive ability (Hampshire et al., 2019).

We make this claim because (a) training interventions may have a strong impact on both task-specific (Engeler & Gilbert, 2020) and domain-general metacognition (Carpenter et al., 2019), and (b) metacognitive interventions have been shown to influence intention offloading strategies that are themselves highly effective (Gilbert et al., 2020, Experiment 3). However, we need to insert two caveats. The first is that the effects of metacognitive interventions on cognitive offloading have been inconsistent (Engeler & Gilbert, 2020; Grinschgl et al., 2020), so it is not well understood why the impact of these interventions varies between studies. The second is that the cross-domain organisation of metacognition and cognitive offloading is still poorly understood. This cross-domain organisation is important because it may determine the extent to which an intervention delivered in one domain would generalise to another. This in turn would determine whether a single intervention could be delivered to influence an individual’s offloading strategies across multiple domains, or whether multiple task-specific interventions would instead be necessary.

## Open Questions

We note here three open questions about intention offloading (and cognitive offloading more generally) that we think are particularly pressing. The first of these concerns the cross-domain organisation of metacognition and cognitive offloading. As noted above, this issue is important for both theoretical and practical reasons. The current evidence is mixed. Gilbert (2015b) provided evidence for a domain-general metacognitive component to intention offloading: Participants’ confidence in a perceptual judgement task correlated with their propensity for intention offloading in a separate memory task. Importantly, the perceptual judgement task in this experiment used a staircase procedure, so that accuracy was equalised between participants. This means that variation in perceptual confidence had no relationship with objective accuracy. Nevertheless, this variation in perceptual confidence – a measure of metacognitive bias – was associated with intention offloading in a separate task. This suggests that a metacognitive intervention in one domain (e.g., perception) may affect cognitive offloading in a different domain (e.g., memory).

In a follow-up study, Sachdeva and Gilbert (in preparation) replicated the finding that participants’ perceptual confidence was related to their propensity for intention offloading in a separate task. However, perceptual confidence was not significantly associated with individuals’ *bias* towards or away from reminders, relative to the optimal strategy. Therefore, while evidence suggests that there is some domain-general component of the metacognition-offloading link, it is not known how far this extends to individuals’ *preference* for offloading, relative to the optimal strategy, compared with their *propensity* for offloading, which may also reflect their level of cognitive ability (and hence the need for offloading) regardless of preference.

Studies investigating the relationship between propensity for cognitive offloading in different domains have also produced inconsistent results. Ball et al. (2022) found that intention offloading strategies were significantly correlated between three tasks using different stimulus materials. Specifically, the rate of offloading was significantly correlated across the three tasks but the *bias* in each task, relative to the optimal strategy, was not. On the other hand, Meyerhoff et al. (2021) did not find any significant association between cognitive offloading strategies measured in an intention offloading task and a perceptual block-copy task. In sum, we know relatively little about domain-specific versus domain-general contributions to cognitive offloading strategies, and the impact of metacognitive training on those strategies. If we are to develop real-world metacognitive interventions to improve individuals’ cognitive offloading strategies, we need to make progress on both of these issues.

The second open question that we highlight concerns the relationship between cognitive offloading, as measured in experimental tasks like the ones described above, and people’s everyday practice of cognitive offloading in the real world. Undoubtedly, the complexities of individuals’ decisions about offloading in the real world, and the factors influencing those decisions, will not be fully captured by our experimental tasks. For example, the studies described in this review have paid little attention to the factors that vary between different types of reminder, such as how effortful they are to set up, what demands are offloaded (e.g., attentional vs. memory demands), and how effective they are (or how effective they are believed to be; Weis & Wiese, 2019). As well as investigating these factors in the laboratory, experimental methods could be complemented by more naturalistic approaches such as diary studies or observation of naturalistic behaviour (Rummel & Kvavilashvili, 2019).

The final open question that we highlight concerns individuals’ motivations for engaging in cognitive offloading. What factors do people weigh up when they decide whether to offload? These factors could serve as potential targets for interventions to improve cognitive offloading strategies. The answer implicit in much of the discussion above is that we offload in order to increase performance. For example, a person might engage in intention offloading in order to increase the probability of remembering. However, this is probably not the only factor that individuals consider. Another possibility is that we offload, not so much to increase performance, but to reduce uncertainty. Once an intention has been offloaded, there is probably less uncertainty in whether or not it will actually be realised. This in turn can improve the stability, consistency, and predictability of behaviour. Therefore, an open question for future research is whether the performance-increasing and the uncertainty-reducing consequences of offloading can be distinguished from each other.

A further possible motivation for offloading could be that by offloading one cognitive process, individuals are then better able to engage in another. For example, saving one list of to-be-remembered words to an external store can improve memory for a subsequently presented list (Storm & Stone, 2014) or even performance of an unrelated task (Runge et al., 2019). Similarly, Dupont et al. (in press) found that offloading high-value intentions to external reminders led to a ‘spillover’ effect whereby internal memory was reallocated to remaining low-value intentions (see also Risko et al. (in press) for discussion of a related point). Therefore, individuals might engage in offloading not to improve performance or reduce uncertainty for the offloaded task, but instead for the cognitive savings that this can engender for a subsequent activity. To the extent that individuals are motivated by these different factors to engage in offloading, different interventions might be required to alter their strategies.

## Conclusion

We hope that the work summarised in this review can provide a basis for designing interventions to influence cognitive offloading strategies. However, there is clearly still a gulf between the experimental studies reviewed above and the design of real-world interventions. Closing this gap could increase the adaptive use of technology and support individuals’ ability to live healthy, independent and purposeful lives.
